# Global Transcriptome Sequencing Reveals Molecular Profiles of Summer Diapause Induction Stage of Onion Maggot, *Delia antiqua* (Diptera: Anthomyiidae)

**DOI:** 10.1534/g3.117.300393

**Published:** 2017-11-20

**Authors:** Shuang Ren, You-Jin Hao, Bin Chen, You-Ping Yin

**Affiliations:** * Key Lab of Genetic Function and Regulation in Chongqing, School of Life Sciences, Chongqing University, 401331, China; † Institute of Entomology and Molecular Biology, College of Life Sciences, Chongqing Normal University, 401331, China

**Keywords:** *Delia antiqua*, transcriptome sequencing, diapause induction, genes and pathways, circadian clock

## Abstract

The onion maggot, *Delia antiqua*, is a worldwide subterranean pest and can enter diapause during the summer and winter seasons. The molecular regulation of the ontogenesis transition remains largely unknown. Here we used high-throughput RNA sequencing to identify candidate genes and processes linked to summer diapause (SD) induction by comparing the transcriptome differences between the most sensitive larval developmental stage of SD and nondiapause (ND). Nine pairwise comparisons were performed, and significantly differentially regulated transcripts were identified. Several functional terms related to lipid, carbohydrate, and energy metabolism, environmental adaption, immune response, and aging were enriched during the most sensitive SD induction period. A subset of genes, including circadian clock genes, were expressed differentially under diapause induction conditions, and there was much more variation in the most sensitive period of ND- than SD-destined larvae. These expression variations probably resulted in a deep restructuring of metabolic pathways. Potential regulatory elements of SD induction including genes related to lipid, carbohydrate, energy metabolism, and environmental adaption. Collectively, our results suggest the circadian clock is one of the key drivers for integrating environmental signals into the SD induction. Our transcriptome analysis provides insight into the fundamental role of the circadian clock in SD induction in this important model insect species, and contributes to the in-depth elucidation of the molecular regulation mechanism of insect diapause induction.

The onion maggot, *Delia antiqua*, is a serious pest of onion, *Allium cepa*, and is widely distributed over Asia, Europe, and North America (Ishikawa *et al.* 2000). In the laboratory, summer and winter diapause of this species are induced by high temperature/long day length and low temperature/short day length, respectively (Ishikawa *et al.* 1987; Chen *et al.* 2010). During summer diapause (SD) induction, the most sensitive larval developmental stage for temperature with regard to diapause induction was estimated to be between pupariation and “pupation” (Ishikawa *et al.* 2000). Until this age, all pupae alter their developmental destiny from direct development to diapause. Some aspects of diapause are well established in this species, for instance, RNA sequencing (RNA-Seq) was applied to investigate the genome-wide gene expression differences between nondiapause (ND) and SD pupae (Zhang *et al.* 2014; Hao *et al.* 2016). However, little work has been performed to elucidate the molecular basis of diapause induction in this species.

Diapause is an alternative life history strategy and a physiologically dynamic developmental pathway coordinated by a suite of neuroendocrine and metabolic controls (Denlinger 2002). Many studies of the synchrony between seasonal events and insect life histories have focused on insect diapause; however, molecular regulation of diapause remains largely unknown because of the complexity of the diapause phenotype (Meuti *et al.* 2001; Kostál *et al.* 2009; Ikeno *et al.* 2010, 2011; Kankare *et al.* 2010; MacRae 2010; Yamada and Yamamoto 2011). Moreover, diapause is an alternative, dynamic pathway to normal active development, with various phases and modules (Kostál 2006; Emerson *et al.* 2009). Characterizing the physiological processes in the underlying regulatory mechanism of the seasonal timing of diapause remains a central challenge. Our understanding of the molecular mechanisms of insect diapause has expanded rapidly, with most studies focusing on comparisons of diapausing and nondiapausing individuals, thereby reflecting important control mechanisms (Flannagan *et al.* 1998; Daibo *et al.* 2001; Yocum 2003; Robich and Denlinger 2005; Robich *et al.* 2007; Sim and Denlinger 2009; Kankare *et al.* 2010; MacRae 2010; Ragland *et al.* 2010; Rinehart *et al.* 2010; Poelchau *et al.* 2011, 2013; Dong *et al.* 2014; Qi *et al.* 2015; Hao *et al.* 2016; Kang *et al.* 2016). Despite increasing knowledge of diapause preparation, initiation, maintenance, and termination, molecular mechanisms regulating the more “upstream” stage of diapause induction remain largely unexplored. Diapause induction occurs during a genotype-specific ontogenetic stage (sensitive period), when environmental cues are perceived and transduced into switching the ontogenetic pathway from direct development to diapause when the token stimuli reach some critical level (Kostál 2006). Many studies were conducted to reveal candidate genes and processes that regulate insect prediapause (Kostál *et al.* 2009; Reynolds and Hand 2009; Trionnaire *et al.* 2009; Urbanski *et al.* 2010; Reynolds *et al.* 2013; Poelchau *et al.* 2011, 2013; Hickner *et al.* 2015; Huang *et al.* 2015; Poupardin *et al.* 2015). Among these studies, only very few focused on diapause induction, and the molecular mechanisms regulating diapause induction remain much less well understood (Kostál *et al.* 2009; Reynolds *et al.* 2013; Hickner *et al.* 2015; Huang *et al.* 2015; Poupardin *et al.* 2015). Insect species enter diapause in different ontogenetic stages. Phenotypic features of diapause induction are also different among most insect species. There may be diverse transcriptional strategies for producing them. Previous studies used transcriptomic technologies to characterize gene expression changes in response to diapause induction to reveal specific genes and processes that critically regulated this development transition. Transcriptional evidence for small RNA (sRNA) regulation of pupal diapause of the flesh fly, *Sarcophaga bullata*, indicated a role for sRNA in programming the switch from direct development to diapause (Reynolds *et al.* 2013). A global pattern of gene expression associated with very early stages of diapause indicated that short day triggering of diapause was associated with inhibition of 20-HE signaling during the photoperiod-sensitive period of larvae of the drosophilid fly *Chymomyza costata* (Poupardin *et al.* 2015). Whole-transcriptome microarrays revealed some potential regulatory mechanisms driving diapause induction of *Culex pipiens* female adults, including the TGF-β and Wnt signaling pathways, ecdysone synthesis, chromatin modification, and the circadian rhythm (Hickner *et al.* 2015). In nonblood-fed female adults of *Aedes albopictus*, potential regulatory elements of diapause induction include two canonical circadian clock genes, *timeless* and *cryptochrome1*, while in blood-fed females, genes related to energy production and offspring provisioning were differentially expressed, including oxidative phosphorylation pathway and lipid metabolism genes (Huang *et al.* 2015). In this study, we identified the differentially expressed genes (DEGs) during the most sensitive larval developmental period (9-d-old and 4-d-old third-instar larvae reared under SD- and ND-destined conditions, respectively) of *D. antiqua*. Our study not only provides information about potential regulation components of diapause induction but also provides insight into the influence of rearing temperature on the switch between two alternative development pathways. Diapause induction-associated functions of candidate genes can be further interrogated using functional genomics approaches in *D. antiqua* and other insect species.

## Materials and Methods

### Insect rearing, diapause induction, and sampling

**Figure 1:**
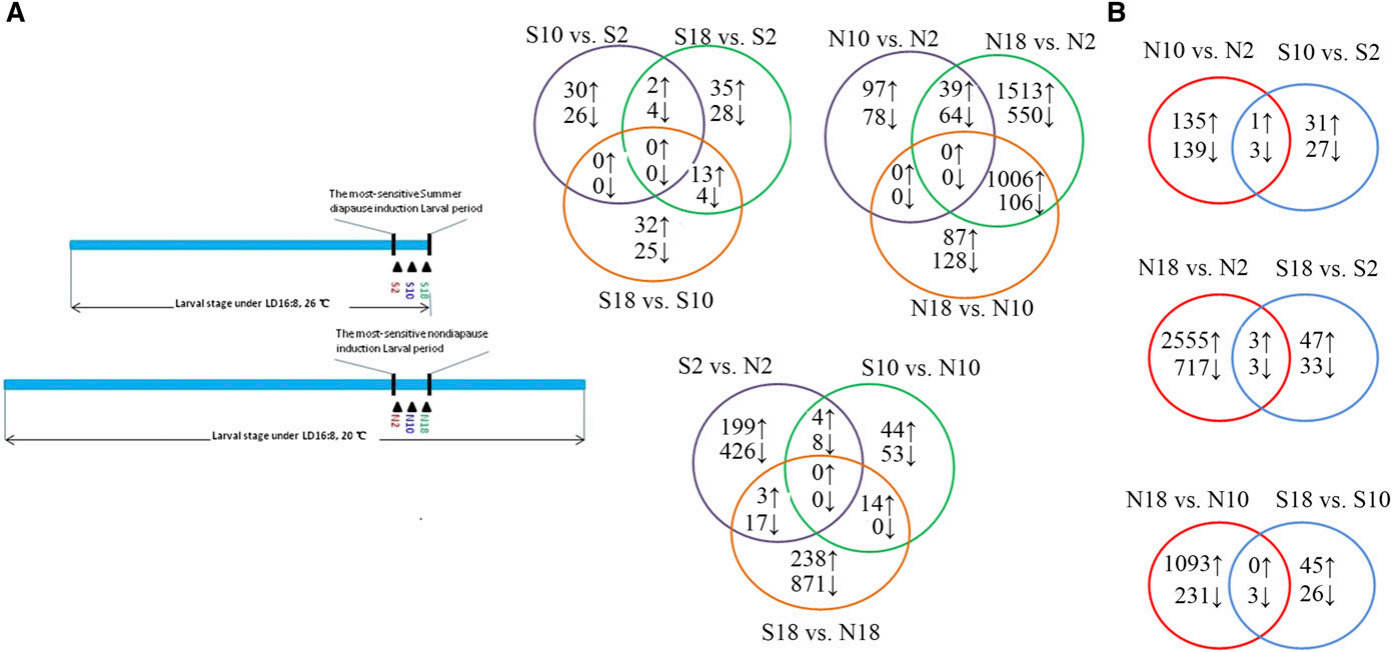
Comparisons among transcripts during sensitive period of ND- and SD-destined larvae of *D. antiqua*. (A) Experimental design. The larvae were sampled at three different Zeitgeber times: N2 (S2), 4 hr after lights off; N10 (S10), 4 hr after lights on; and N18 (S18), 4 hr before lights off; (B) Venn diagrams showing overlaps among genes with increased (↑, upregulated) or decreased (↓, downregulated) transcript abundance in all pairs of comparisons.

A laboratory *D. antiqua* colony was reared in our laboratory as described previously (Chen *et al.* 2010). ND- and SD-destined larvae were reared at 20 ± 0.5°, 16:8 hr light/dark cycle and 26 ± 0.5°, 16:8 hr light/dark cycle, 50–70% relative humidity, respectively. Periodic interruption experiments of three biological replicates were conducted to determine the sensitive period of diapause induction. Eggs and larvae reared under SD-inducing conditions of 26 ± 0.5°, 16:8 hr light/dark cycle, were transferred to diapause-averting conditions of 20 ± 0.5°, 16:8 hr light/dark cycle, day by day (designated D0 for larva hatching). Diapause incidence increased significantly at D14. Meanwhile, eggs and larvae reared under 20 ± 0.5°, 16:8 hr light/dark cycle, were transferred to 26 ± 0.5°, 16:8 hr light/dark cycle, day by day. Diapause incidence increased significantly at D13. The most sensitive periods of SD- and ND-destined larvae were the 14-d-old larvae (corresponding to 9-d-old third instar) and the 13-d-old larvae (corresponding to 4-d-old third instar), respectively. Potential effects of diurnal oscillation on gene expression were partially controlled by sampling larvae at three *zeitgeber* times corresponding to 4 hr after light on (10:00), 12 hr after light on (18:00), and 4 hr after light off (2:00) (Figure 1A). Samples were prepared from larvae collected at 2:00, 10:00, and 18:00 of 9-d-old and 4-d-old third-instar larvae reared under 26 ± 0.5°, 16:8 hr light/dark cycle; and 20 ± 0.5°, 16:8 hr light/dark cycle, respectively. Samples were frozen in liquid nitrogen and stored at −80° until RNA extraction.

### RNA isolation, library construction, and Illumina sequencing

Total RNA was extracted from pools of five larval heads using a TRIzol RNA extraction kit (Invitrogen, Carlsbad, CA). RNA concentration and integrity were determined by an RNA Nano 6000 Assay Kit and the Agilent Bioanalyzer 2100 system (Agilent Technologies, Santa Clara, CA). The isolated RNA pellets were stored at −80° until needed. Two biological replicates were used in the analysis. Six RNA-Seq libraries were generated using a NEBNextUltra RNA Library Prep Kit (NEB) following the manufacturer’s recommendations. Clustering of the index-coded samples was performed on a cBot Cluster Generation System using a TruSeq PE Cluster Kit v3-cBot-HS (Illumina) according to the manufacturer’s instructions. After cluster generation, the library preparations were sequenced on an Illumina HiSequation 2000 platform, and paired-end reads were generated (read length = 150 bp) (Illumina Inc., San Diego, CA).

### Raw data collection, assembly, and annotation

The raw reads were trimmed and quality controlled by SeqPrep (https://github.com/jstjohn/SeqPrep) and Sickle (https://github.com/najoshi/sickle). Duplicates were removed using SEAL (Pireddu *et al.* 2011). After *de novo* assembly with Trinity (Grabherr *et al.* 2011) with min_kmer_cov 2, min_contig_length 200, min_glue 3, and seqType fq, assembled unigenes were used for a basic local alignment search tool (BLAST) search and annotated against the nonredundant (nr) database at the National Center for Biotechnology Information with an *E*-value cut-off of 1e^−5^.

### DEG analysis

Clean data were mapped back to the assembled transcriptome and read counts for each gene were obtained from the mapping to estimate gene expression level by RSEM (RNA-Seq by Expectation-Maximization) with default parameters (Li and Dewey 2011). R package *DESeq* (version 1.18.0) was used to identify the DEGs between the two groups.

DESeq provide statistical principles for determining differential expression in digital gene expression data using a model based on the negative binomial distribution. The resulting false discovery rate (FDR) was calculated by Benjamini and Hochberg’s algorithm. Genes with an adjusted FDR < 0.05 and |log_2_ ratio| ≥ 1 were considered as differentially expressed.

### Function and pathway enrichment analysis

Based on the results of the protein annotation, the BLAST2GO program (http://www.blast2go.com/b2ghome) with default parameters was employed to obtain the functional classification of the unigenes based on gene ontology (GO) terms. The retrieved GO terms were allocated to query sequences and the genes present in the transcriptome were classified into cellular component, molecular function, and biological process categories. The WEGO (http://wego.genomics.org.cn/cgi-bin/wego/index.pl) was used for functional classification and graphical representation of GO terms. The assembled unigenes were further annotated against the Kyoto Encyclopedia of Genes and Genomes (KEGG) metabolic pathways database (Kanehisa and Goto 2000). We performed an analysis of significantly enriched pathways using a hypergeometric test to detect the significantly enriched pathways of the DEGs.

### Protein–protein interaction (PPI) network construction

PPI network analysis was constructed using the iRefIndex database with default parameters to identify coexpressed gene networks, gene modules, and the hub genes (top connectivity with other genes) in each module (http://www.networkanalyst.ca/) (Xia *et al.* 2015).

### Validation of RNA-Seq results

The expression levels of 10 genes were determined by Real-time quantitative PCR (RT-qPCR) using the SYBR *Premix Ex Taq* II kit (TaKaRa, Dalian, China) according to the manufacturer’s instruction. RNA samples were the same as those used for RNA-Seq. Total RNA was reverse transcribed into cDNA using the PrimeScript RT Master Mix (Perfect Real Time) Kit (TaKaRa, Shiga, Japan). GAPDH and β-tubulin were chosen as endogenous control genes. The primers used for RT-PCR were shown in Supplemental Material, Table S1. Three biological and technical replicates were performed for each treatment. The relative expression ratio of the target genes was determined by Cq values and calculated using the 2^−ΔΔCt^ method.

### Data availability

The clean reads produced in this study have been deposited at DDBJ/EMBL/GenBank Short Read, archived under project number PRJNA393225, BioSample number N1-1-2h: SAMN07326251, N2-1-2h: SAMN07326253, N1-1-10h: SAMN07326289, N2-1-10h: SAMN07326291, N1-1-18h: SAMN07326295, N2-1-18h: SAMN07326300, S1-2-2h: SAMN07326303, S2-1-2h: SAMN07326307, S1-1-10h: SAMN07326309, S2-1-10h: SAMN07326310, S1-1-18h: SAMN07326313, S2-1-18h: SAMN07326314. Accession code SRP111154.

The supplemental files contain the following data. Table S1 contains primers used for the quantitative real time-polymerase chain reaction analysis. Table S2 contains 21 significantly enriched GO terms from DEGs. Table S3 contains the KEGG pathway analysis of DEGs among nine different comparisons. Table S4 shows the genes involved in environmental adaption and the immune system as determined by annotating the *D. antiqua* transcriptome and the DEG analysis. Table S5 shows relative expression levels of various genes of selected signaling pathways in sensitive period of ND- and SD-destined larvae of *D. antiqua*. Table S6 shows the enzymes involved in lipid metabolism as determined by annotating the *D. antiqua* transcriptome and the DEG analysis. Table S7 shows the enzymes involved in carbohydrate metabolism as determined by annotating the *D. antiqua* transcriptome and the DEG analysis. Table S8 shows the enzymes involved in energy metabolism as determined by annotating the *D. antiqua* transcriptome and the DEG analysis. Table S9 contains Cq values, average Cq values (mean Cq), and associated SD (Cq SD) of two endogenous control genes (GAPDH and β-tubulin) and 10 genes of interest.

## Results and Discussion

### Characteristics, functional annotation, and classification of assembled transcripts

**Figure 2:**
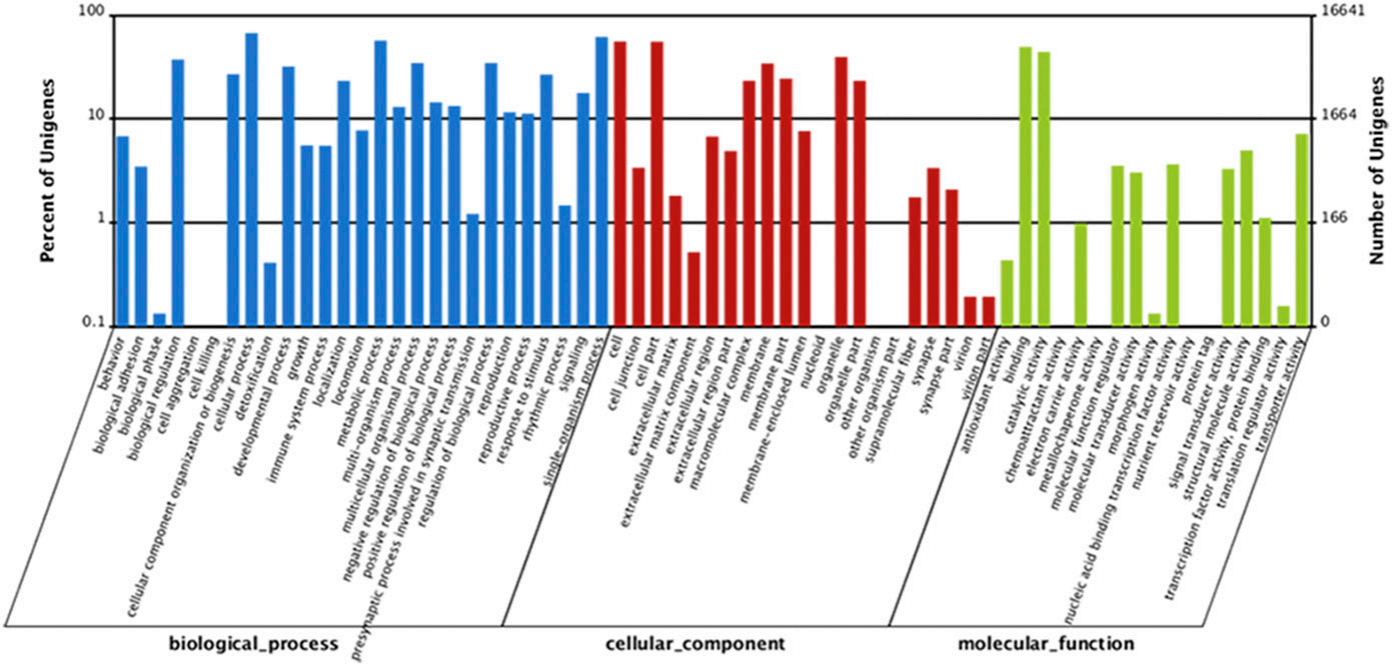
GO classification of *D. antiqua* unigenes.

**Figure 3:**
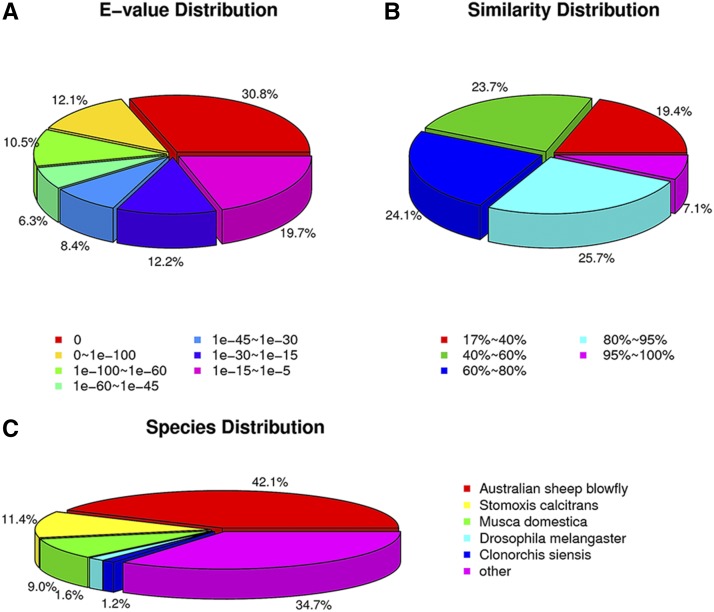
Nonredundant database classification of *D. antiqua* unigenes. (A) *E*-value distribution. (B) Similarity distribution. (C) Species distribution.

A total of 49,337,838 raw reads were generated from the six libraries (Table 1). After removing low-quality sequences and ambiguous nucleotides, 46,589,811 clean reads were obtained. For sequence mapping, the transcriptome reference was reassembled and produced 59,389 unigenes. A total of 16,641 unigenes were grouped into 65 GO functional categories, which were distributed under three categories of Molecular Function (*n* = 17), Biological Process (*n* = 27) and Cellular Components (*n* = 21) (Figure 2). After the nr database annotation, the distributions of *E*-value, identity, and species distribution were analyzed (Figure 3, A–C). For the *E*-value distribution of the predicted proteins, the top hits indicated that 30.8% of the mapped sequences had a significant similarity with a stringent threshold (0) (Figure 3A). For the similarity distribution, 32.8% of the sequences had a similarity higher than 80% (Figure 3B). The species distribution showed that genes from *D. antiqua* had the greatest number of matches with those of the Australian sheep blowfly (Figure 3C).

**Table 1:** Summary of RNA sequencing, assembling, and functional annotation for *D. antiqua*

Sequencing and Assembling	Statistics and Annotation (*E*-value ≤ 1e^−5^)
Raw Reads Number	49,337,838
Clean Reads Number	46,589,811
No. of Total Clean Nucleotides (nt)	6,988,471,675
Q20 Percentage of Total Clean Reads	97.91%
GC Percentage of Total Clean Nucleotides	41.38%
Number of Unigenes	45,281
Total Length (nt) of Total Unigenes	63,044,362
Mean Length (nt) of Total Unigenes	1392
N50 (nt) of Total Unigenes	2443
Unigenes with Nr Database	34,131 (75.4%)
Unigenes with Nt Database	22,680 (50.1%)
Unigenes with Swiss-Prot Database	26,320 (58.1%)
Unigenes with KEGG Database	24,277 (53.6%), 257 pathways
Unigenes with COG Database	15,791 (34.9%), 25 functional categories
Unigenes with GO Database	16,641 (36.8%), 65 subcategories belonging to three main categories
Biological Process	27 subcategories
Cellular Component	21 subcategories
Molecular Function	17 subcategories
Total Unigenes Annotated	36,610 (80.9% of 45,281)

### Detection of DEGs and GO classification

Six digital gene expression libraries were constructed to provide comprehensive gene expression profiling between the ND and SD induction periods (Figure 1A). Nine pairwise comparisons were performed to investigate the expression patterns of genes with fold change ≥ 2.0 and *P*-value < 0.05. Results showed that dramatic changes occurred at the sensitive period of ND-destined larvae (Figure 1B).

Dominant GO terms of the biological process subcategories were grouped into cellular processes and single-organism processes. Among 21 subcategories of cellular components, assignments were mostly given to cell and cell part. Within the molecular function category, there were high percentages of genes with binding and catalytic activities. The top 21 significantly enriched GO terms from DEGs were generated for eight genes (3.6%) in S2 *vs.* N2, 36 genes (12.4%) in S18 *vs.* N18, 378 genes (46.8%) in N18 *vs.* N2 (113 genes were mapped to oxidoreductase activity), and 118 genes (36.5%) in N18 *vs.* N10 (54 genes were mapped to structural molecule activity), respectively (Table S2). Results showed that diverse structural, regulatory, metabolic, and transporter proteins were encoded by expressed genes in *D. antiqua*.

### Unigene Clusters of Orthologous Groups (COG) and KEGG pathway assignments

**Figure 4:**
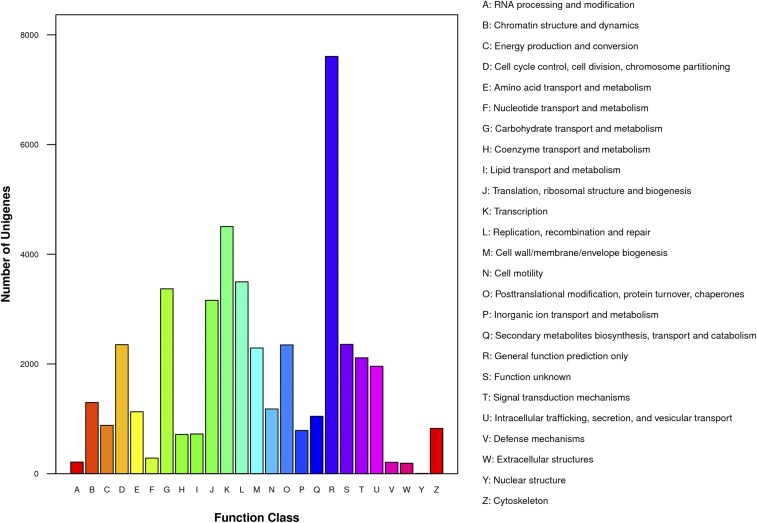
COG classification of *D. antiqua* transcriptome unigenes.

The 45,281 unigenes were searched against the COG database and 15,791 unigenes were classified into 25 functional categories. The three largest categories were general function prediction only; transcription; and replication, recombination, and repair, and the smallest category was extracellular structures (Figure 4). After searching the 45,281 unigenes against the KEGG database, 24,277 unigenes were assigned to 257 KEGG pathways (Table 1).

**Figure 5:**
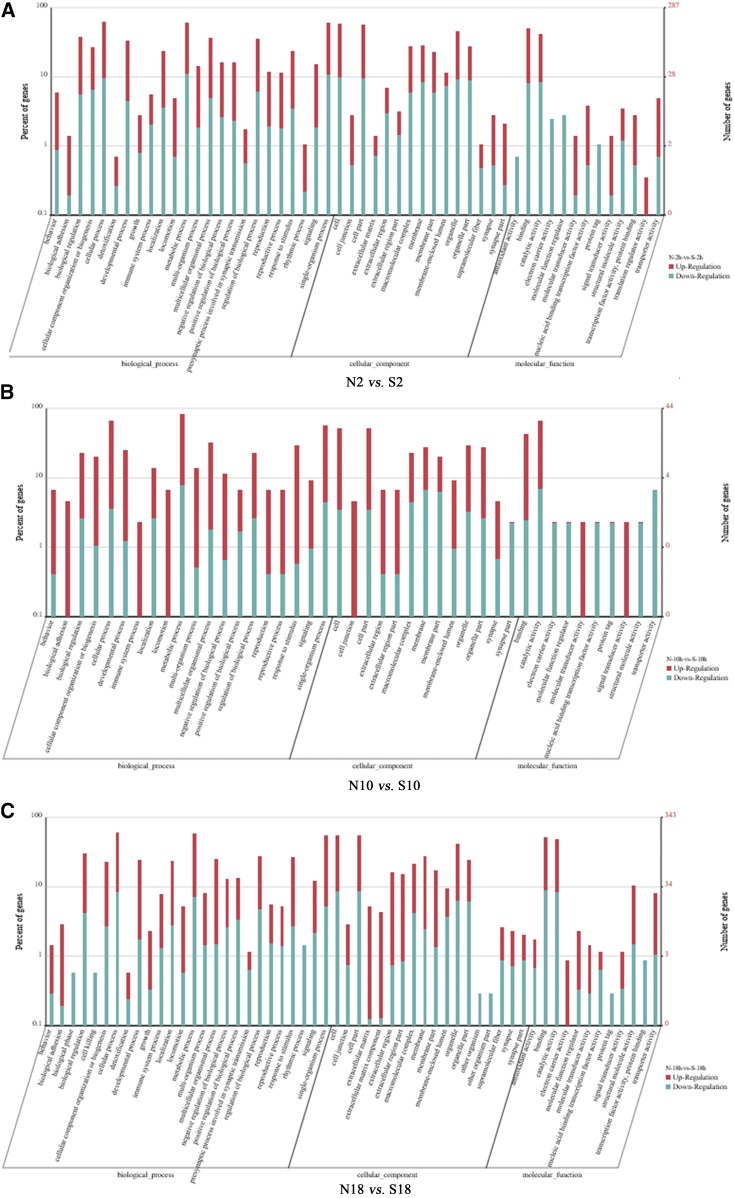
GO annotation of differentially expressed genes in S2 *vs.* N2 (A), S10 *vs.* N10 (B), and S18 *vs.* N18 (C). N2 (S2), 4 hr after lights OFF; N10 (S10), 4 hr after lights ON; and N18 (S18), 4 hr before lights OFF. Left panel, the *y*-axis indicates the percentage of a specific category of unigenes; right panel, the *y*-axis indicates the number of unigenes in a category.

Based on GO annotations, DEGs were assigned to different pathways (Table S3). The “metabolism” was the most highly represented category, which led to in-depth analysis of this group. Several unigenes were associated with lipid metabolism, stress response, oxidative stress protection, innate immune response, and circadian rhythm (Figure 5). Among them, heat shock proteins (HSPs) were significantly overrepresented in the downregulated genes, and circadian clock genes were conserved in insect diapause control. The expression pattern of HSPs and circadian clock genes indicates that they have potential roles in diapause induction of this species.

### Differentially expressed transcripts in relation to SD induction

In SD *vs.* ND, 46 differentially expressed transcripts were shared between the SD and ND regimes. Potential regulatory elements of diapause induction were transcriptionally upregulated, including clock gene *period*, *Mucin-2*, *trypsin*, *chitinase*, multidrug resistance protein 1A, and NADH dehydrogenase. Upregulated transcripts were linked with the circadian period, thermosensitivity, and immune defense. A total of 23 and 1215 transcripts were shared among three different time points of SD and ND induction, respectively (Figure 1B). Genes involved in circadian rhythm, longevity regulation, oxidative phosphorylation, stress response, the tricarboxylic acid (TCA) cycle, fatty acid metabolism, and the mitogen-activated protein kinase (MAPK) signaling pathway were differentially expressed under ND. The circadian clock gene *period* was downregulated 16 hr after light off under ND, and circadian-regulated gene *takeout* was also downregulated 8 and 16 hr after light off under ND. However, casein kinase, *shaggy*, and Skp1-Cullin-F-box complex were all upregulated under ND (Table S4).

### Important signaling pathways

Metabolic pathways including the MAPK, Notch, calcium (Ca^2+^), and vascular endothelial growth factor (VEGF) signaling pathways were enriched during the sensitive period of SD (Table S5). Genes in the MAPK pathway showed significant alterations between ND and SD induction. Growth factor receptor-bound protein 2 is known to transduce activated tyrosine kinase signaling to Ras, which subsequently facilitates the activation of downstream signaling pathways, including Ras and MAPK. GRB2 was downregulated in S2 *vs.* N2, but not in S10 *vs.* N10 or S18 *vs.* N18. Up- and downregulated genes, including GRB2, guanine nucleotide-binding protein subunit α-12, Ras-related protein Rab-40, Ras-related protein Rab-5C, inositol-3-phosphate synthase, cyclin-dependent kinase regulatory subunits, U6 snRNA-associated Sm-like protein LSm6, and Denticleless, were involved in the MAPK pathway during induction of SD, indicating that they are important for SD induction of *D. antiqua*.

The expression patterns of *notch*, *mastermind*, *deltex*, and serrate RNA effector molecule homolog isoform X4 of the Notch signaling pathway were differentially altered during SD induction. *Notch* was significantly downregulated, but *mastermind*, *deltex*, and serrate RNA effector molecule homolog isoform X4 were significantly upregulated in S2 *vs.* N2. The Notch signaling pathway has been shown to have a role in the mature adult brain and in nondevelopmental decisions in *Caenorhabditis elegans*, *Drosophila*, and mice (Costa *et al.* 2003; Chao *et al.* 2004; Yu *et al.* 2008). *Notch* has been shown to be involved in regulating *C. elegans* dauer maintenance and recovery (Ouellet *et al.* 2008); however, its role in insect diapause regulation is still unknown. In *Drosophila*, *mastermind* is widely expressed, overlapping other neurogenic loci during embryonic and postembryonic development, and its transcription is widespread during most of embryogenesis (Schmid *et al.* 1996). An extensive phenotypic characterization of loss-of-function *deltex* phenotypes showed abnormalities suggesting that *deltex* is involved in cell fate decision processes (Gorman and Girton 1992). The differences in transcriptional patterns of genes involved in the Notch signaling pathway indicate a potential role for notch signaling in SD induction in *D. antiqua*.

The VEGF pathway is critical in controlling cell migration during *Drosophila* oogenesis (Cho *et al.* 2002). Two genes, serine/threonine-protein phosphatase 6 regulatory ankyrin repeat subunit B and *calnexin*, were significantly downregulated in S2 *vs.* N2 (Table S5). A multifunctional role for *calnexin* was demonstrated, as both a molecular chaperone uniquely required for rhodopsin maturation and a regulator of Ca^2+^ that enters photoreceptor cells during light stimulation (Rosenbaum *et al.* 2006). *Drosophila calnexin* colocalizes and interacts with sodium channel Paralytic (*Para*), and *calnexin*-mediated *Para* function is required for locomotor behavior and underlies synaptic differentiation (Xiao *et al.* 2017). Therefore, downregulation of *calnexin* may repress transcriptions in relation to SD induction in *D. antiqua*.

Ca^2+^ signals are crucial for the control of a broad range of cellular functions, including secretion, motility, metabolism, growth, cell division, and apoptosis. Many channels, pumps, and cellular proteins have been found to comprise the machinery for generating cellular Ca^2+^ signals. However, it is less well understood how these proteins coordinate during the insect diapausing process. Our study highlighted that *Adenine nucleotide translocator*, *Calmodulin-like protein* 4, and *Calcium/calmodulin-dependent protein kinase* of the Ca^2+^ signaling pathway were significantly downregulated during the induction of SD. Therefore, the Ca^2+^ signaling pathway may be an important component during diapause induction.

### Variation of metabolic pathways during diapause induction

Adjustments of metabolic rates are major adaptive responses to environmental stresses (Robich and Denlinger 2005). The circadian clock organizes metabolic functions in a daily schedule and synchronizes this schedule with environmental rhythms. In this study, 66 genes involved in lipid, carbohydrate, and energy metabolism were enriched, and most of them were expressed rhythmically in ND but not in SD induction, which led to a better understanding of the clock-controlled metabolic regulation in diapause induction (Table S6, Table S7, and Table S8). Genome-wide expression profiling uncovered a potential connection between the circadian clock and metabolic pathways such as energy, carbohydrate, amino acid, lipid, and protein metabolism, and detoxification (Rund *et al.* 2011). Many key rate-limiting steps in anabolic/catabolic metabolic pathways are under circadian or diel control; therefore, clock and clock-controlled gene expression plays a crucial part in metabolic rhythms (Mazzoccoli *et al.* 2012). Pathways such as oxidative phosphorylation, gluconeogenesis, and lipogenesis have been found to be expressed rhythmically (Wijnen and Young 2006; Kohsaka and Bass 2007). In *D. antiqua*, 66 differentially regulated transcripts indicate that a metabolic switch to lipid, carbohydrate, and energy accumulation starts very early, even during the diapause induction stage. Significantly rhythmical expression patterns of genes involved in steroid biosynthesis, fatty acid metabolism, the citrate cycle, oxidative phosphorylation, and stress response indicated potential involvement of the circadian clock in SD induction in *D. antiqua*.

### Lipid metabolism

To gain insights into distinct lipid metabolism during the diapause induction, the lipid metabolism-related genes were analyzed. Genes encoding Δ14-sterol reductase, sterol-4α-carboxylate 3-dehydrogenase, triacylglycerol lipase, and fatty acid synthase were downregulated during the induction of SD. Genes encoding triacylglycerol lipases, elongases, desaturases, and lipophorin receptors were significantly downregulated in SD when compared with ND (Table S6). These patterns indicated a metabolic switch during diapause induction, but there was no functional explanation of a single DEG associated with steroid biosynthesis during these stages.

### The TCA cycle and amino sugar and nucleotide sugar metabolism

Cross talk between the brain and fat body as a regulator of diapause suggested that the TCA cycle may be a checkpoint for regulating insect diapause (Xu *et al.* 2012). Genes encoding citrate synthase, isocitrate dehydrogenase, ATP citrate (pro-S)-lyase, aconitate hydratase, and isocitrate dehydrogenase were downregulated during SD induction. Genes encoding chitinase, ManA, UGDH, and GMPP, which are involved in amino sugar and nucleotide sugar metabolism, were differentially regulated during SD induction (Table S7).

### HSPs

Insect HSP, a ubiquitous component of the stress response in diverse organisms, increases in response to temperature extremes, crowding, starvation, and hypoxia/ anoxia, and the functions of individual proteins within the HSP family often differ with developmental stage, subcellular location, and environmental conditions (King and MacRae 2015). Many HSPs are upregulated under thermal stress; therefore, they represent an important, inducible chaperone family involved in stress response (Li *et al.* 2007; MacRae 2010; King and MacRae 2015). The differential synthesis of HSPs and their responsibility for protein folding and storage, as influenced by ATP, predict their role in protein maintenance and stress tolerance throughout diapause. During diapause, the expression of HSP genes and the accumulation of their products undergo differential regulation, and the association of diapause and HSPs has been documented in several insect species (Chen *et al.* 2006; Rinehart *et al.* 2007; Tungjitwitayakul *et al.* 2008; Gkouvitsas *et al.* 2009). In the present study, genes encoding 16 HSPs were downregulated in SD induction when compared with ND (Table S8). This is in accordance with the observation that transcription of a typical HSP is known to have very rapid response dynamism under stress. A total of 13 HSPs mRNA are substantially downregulated in SD-destined larvae and oscillate in ND-destined larvae. By contrast, HSP23, HSP67B2, and HSP67B3 mRNAs change only relative slightly and oscillate in SD-destined larvae. These results revealed that temperature could influence the expression profile of HSPs, and that they may be involved in reducing the ecological challenges posed by environmental stress. Each HSP may play a specific part in the rapid increase in stress tolerance noted during diapause induction in this species. The coordinated downturn of HSP mRNA suggests that these HSPs cooperate to protect larvae from diapause-associated stress. It was unclear whether, and to what extent, these proteins were necessary to trigger the diapause responses. More data will be needed to draw a conclusion regarding the activity of HSPs in diapause induction.

### Candidate genes involved in the circadian clock

To date, several circadian clock genes in *Drosophila* have been shown to participate in the transcription-translation feedback loops that comprise the central oscillator controlling overt behaviors and have been implicated in the initiation of photoperiodic diapause in several insect species (Goto and Denlinger 2002; Shimada *et al.* 2008; Ikeno *et al.* 2011; Meuti *et al.* 2015). In adult *Drosophila* heads, hundreds of transcripts are under the control of circadian clock genes, and a subset of genes involved in learning and memory/synapse function, vision, olfaction, locomotion, detoxification, and metabolism showed particularly robust cycling and many oscillatory phases (Claridge-Chang *et al.* 2001). Expression patterns of *period* under the same mean temperature and different amplitudes suggest that the mechanism that responds to temperature amplitude involves the circadian clock (Miyazaki *et al.* 2016). The clock genes *casein kinase I isoform epsilon-like*, *casein kinase II*, period, *takeout*, *casein kinase I isoform delta-A-like isoform X3*, *shaggy*, and *Skp1 Cdc53 F-box complex* were significantly differentially expressed in ND, but not in SD induction (Table S4). *Period*, an essential component of the circadian clock system in *Drosophila*, was upregulated under SD induction. This distinct expression pattern of the clock genes suggests that this information could be used to determine alternative developmental strategies such as diapause.

**Figure 6:**
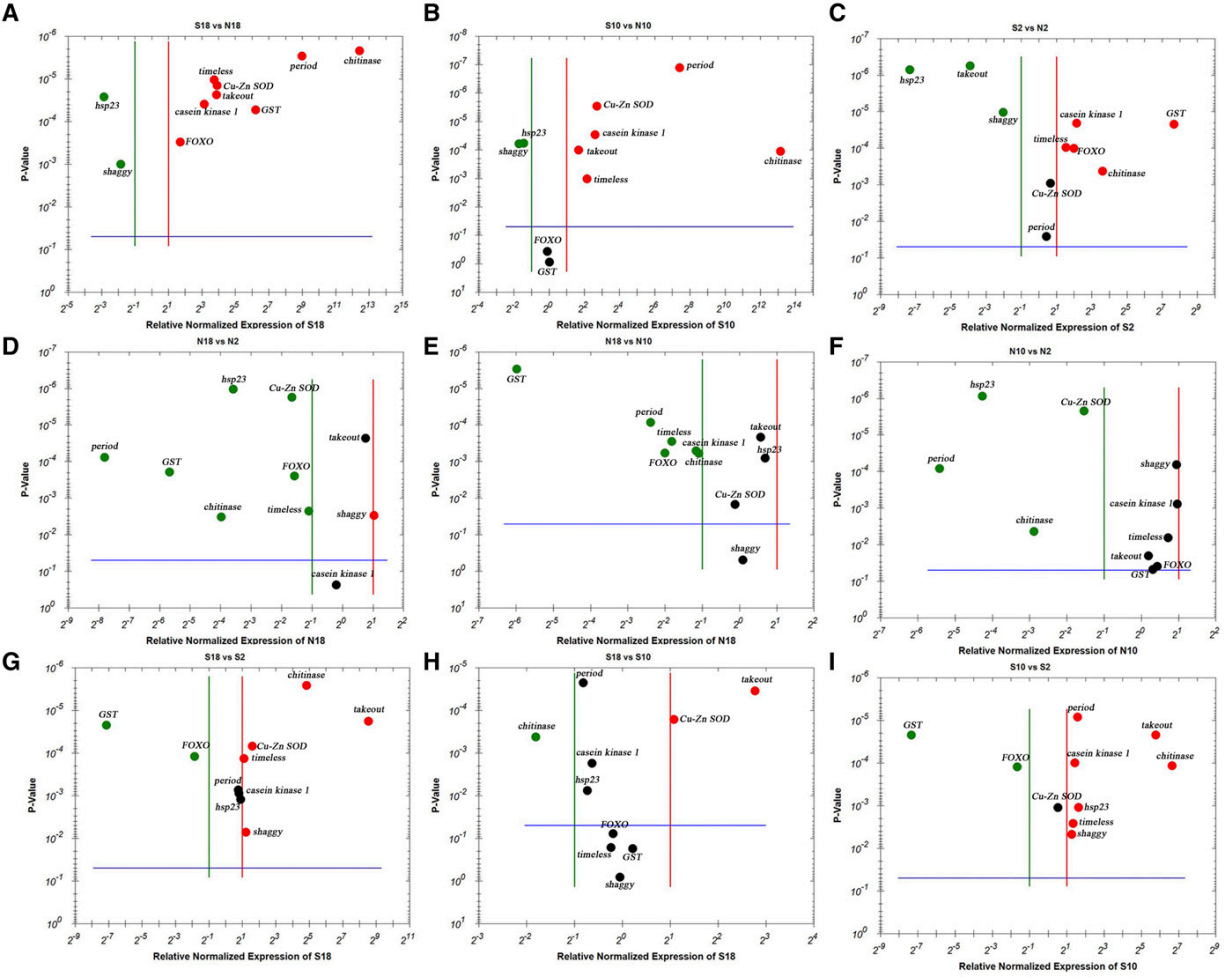
Volcano plot representing significance of genes at three different time points of sensitive period of ND- and SD-destined larvae of *D. antiqua* with *P* < 0.05. The upregulated genes are shown in red, downregulated genes are shown in green, while no significant change genes are shown in black. (A) S18 *vs.* N18; (B) S10 *vs.* N10; (C) S2 *vs.* N2; (D) N18 *vs.* N2; (E) N18 *vs.* N10; (F) N10 *vs.* N2; (G) S18 *vs.* S2; (H) S18 *vs.* S10; (I) S10 *vs.* S2.

*Period*, *shaggy*, *takeout*, *casein kinase I*, and *timeless* were confirmed to be differentially expressed by qRT-PCR (Figure 6). A great number and diversity of genes are expressed in a circadian manner in the predicted molecular clock model of *Drosophila*. A subset of these genes, including those encoding clock components, were differentially expressed between the sensitive period of ND- and SD-destined larvae, suggesting that the expression differences were involved in diapause induction of *D. antiqua*. However, a clear connection between these events has yet to be demonstrated.

### Stress responses

Variations in glutathione S-transferase activity in the eggs of *Bombyx mori* were closely related to embryonic diapause induction and termination in the silkworm (Meng 2010). Ferritin is associated with a stress response during the early diapause of *Nasonia vitripennis* (Wolschin and Gadau 2009) and *Helicoverpa armigera* (Zhang *et al.* 2013). As ferritin is an important iron-scavenging protein, its accumulation during the sensitive period of SD can prevent destructive oxidative stress triggered by free iron. DAF-9/cytochrome P450 produced hormonal signals to regulate *C. elegans* dauer in response to environmental cues (Gerisch and Antebi 2004). Genes encoding glutathione S-transferases and ferritin were significantly upregulated during the sensitive period of SD induction, while *P450*, *catalase*, and *peroxiredoxin* decreased during this period. The expression patterns of these genes suggested their potential role in the regulation of the diapause induction in *D. antiqua*.

Activation of forkhead transcription factor (FOXO) is critical for fat accumulation, cessation of ovarian development, and the elevated antioxidant responses associated with diapause in *C. pipiens* (Sim and Denlinger 2008, 2009, 2013). FOXO emerges as a prime candidate for activating many of the diverse physiological pathways that generate the diapause phenotype. Genes targeted by FOXO are linked to pathways important for diapause, such as stress tolerance, energy storage, lifespan extension, cell cycle regulation, and circadian rhythms. The FOXO transcript was upregulated under SD-inducing conditions. FOXO was differentially expressed under SD-inducing conditions, suggesting that it could be a regulatory component of SD induction in *D. antiqua*.

### Hub gene identification

Gene network analysis revealed potential regulatory elements of SD induction in *D. antiqua*. The hub genes *deltex*, *hsp23*, *hsp60*, *hsp83*, *Jra*, *COX2*, *period*, *mastermind*, and *shaggy* might play a vital part in diapause induction regulation; further experimental studies are necessary to validate these results.

### Conclusions

In temperate zone, insects take photoperiod as a useful ecological cue in the fall, when decreasing photoperiod signals the approaching winter. During the spring, the developing larvae are subjected to increasingly hot conditions, especially for pupation in the soil. Such conditions probably serve as a cue for entry into SD. In *D. antiqua*, temperature is the primary factor in SD induction, while photoperiod plays an important part in winter diapause induction (Ishikawa *et al.* 1987). In this study, samples collected at two different temperatures (20 and 26°) were used to compare transcriptional differences between SD- and ND-destined larvae. Although the causal relationship between regulation of these phenomena and temperature connected with diapause programming is yet indistinct, it is still a meaningful indicator that the effect is due to diapause programming rather than temperature. Since circadian effects on gene expression were controlled by collecting samples at the same Zeitgeber time, the altered expression of genes under ND- relative to SD-induction conditions can also be interpreted as a response to diapause programming rather than differences in rearing temperature.

Using RNA-Seq and DEG analysis, we identified genes involved in diverse biological or molecular pathways, including genes involved in metabolism, signal transduction, stress response, detoxification, oxidative stress protection, and innate immune response. The transcriptomic profiles were altered very rapidly at different time points in ND-destined larvae. These changes probably resulted in a blockage of direct development and a deep restructuring of metabolic pathways, as indicated by their differential expression. Some candidate genes were revealed as potential regulators of SD induction in *D. antiqua*. A total of 46 differentially expressed transcripts were shared between the SD and ND regimes. Upregulated transcripts were linked with the circadian rhythms, thermosensitivity, and immune defense. These results suggest a potential relationship between circadian clock genes and SD induction in *D. antiqua*, and provide a valuable resource for studying specific processes, functions, and pathways in onion maggot diapause. Identification of hub genes by PPI network analysis provides targets for functional study aimed at developing novel control strategies designed to disrupt the diapause response, a crucial ecological adaptation in a wide range of pest species.

## Supplementary Material

Supplemental material is available online at www.g3journal.org/lookup/suppl/doi:10.1534/g3.117.300393/-/DC1.
