# Supplementary material for: Global Transcriptome Sequencing Reveals Molecular Profiles of Summer Diapause Induction Stage of Onion Maggot, Delia antiqua (Diptera: Anthomyiidae)
Source: G3 (Bethesda). 2017 Nov 20;8(1):207–17. doi: 10.1534/g3.117.300393 (PMC5765349; doi:10.1534/g3.117.300393)
Supplement: Supplementary file 1 [file 207TableS1.docx]

**Table S1 Primers used for qRT-PCR analysis**

| **Primers name** | **Homologues** | **Sequence (5’-3’)** |
| --- | --- | --- |
| Unigene10761_All F | *timeless* | TGGAACAACGAGCAGGCTACA |
| Unigene10761_All R |  | CAACGGTCCTAGCTTCTTAGCCA |
| CL5011.Contig2_All F | *chitinase* | ATGGCTGGATTTGGATAGTGG |
| CL5011.Contig2_All R |  | ATAAAGTTCAATGTGGATGTT |
| Unigene7788_All F | *Cu-Zn superoxide dismutase* | TAGAGCGACGAGTAATAGATA |
| Unigene7788_All R |  | TGAGTAAGAGCACAGGAAATG |
| CL492.Contig1_All F | *hsp23* | CTTTCCTCGGATGGCGTCCT |
| CL492.Contig1_All R |  | TCCTGCTGTTGGTCACCACT |
| CL4974.Contig3_All F | *CK1* | TTTAGGGCTGGATGGCCCTT |
| CL4974.Contig3_All R |  | GCAACTGCCCTCCTGGATGA |
| CL5922.Contig3_All F | *glutathione S-transferase* | CGGTAAGAACGACTCATTGTA |
| CL5922.Contig3_All R |  | AGCGTATTGTTGACCCTCCA |
| Unigene10193_All F | *period* | TTTCCAACCATCTAACAGTCA |
| Unigene10193_All R |  | TCTAACCGTAACCTTCTTTCT |
| CL1746.Contig1_All F | *takeout* | TTATCGTGAAATACGGGGA |
| CL1746.Contig1_All R |  | TCAGCGAGACAAACAACCTAC |
| Unigene4265_All F | *Forkhead box protein O* | AACCACAGACGAGAGCCAGAT |
| Unigene4265_All R |  | ACCCCAAGCATTACGACGC |
| CL4915.Contig1_All F | *shaggy* | TTGGAGGTTTGTGATGCGG |
| CL4915.Contig1_All R |  | GCTGGTGGTGAGGGAGGTG |
| GAPDH F |  | ACGTGGTGCTGCCCAAAACATCATT |
| GAPDH R |  | GGCGGACAGTCAAATCAACAAGG |
| β-Tubulin F |  | TGGCGTGCTGCATGTTGTATCGT |
| β-Tubulin R |  | GCCTGGCACAACAGTTGGTGGT |
