# Supplementary material for: Global Transcriptome Sequencing Reveals Molecular Profiles of Summer Diapause Induction Stage of Onion Maggot, Delia antiqua (Diptera: Anthomyiidae)
Source: G3 (Bethesda). 2017 Nov 20;8(1):207–17. doi: 10.1534/g3.117.300393 (PMC5765349; doi:10.1534/g3.117.300393)
Supplement: Supplementary file 2 [file 207TableS2.docx]

**Table S2** [**21 significantly enriched Gene Ontology (GO) terms from DEGs**](http://figshare.com/articles/_The_top_10_most_significantly_enriched_Gene_Ontology_GO_terms_from_differentially_expressed_genes_DEGs_in_Yorkshire_pigs_/1561621).

| **No.** | **GO term** | **Description** | **Corrected *P* value** | **Cluster frequency** |  |
| --- | --- | --- | --- | --- | --- |
| 1 | GO:0005198 | [structural molecule activity](http://amigo.geneontology.org/cgi-bin/amigo/go.cgi?action=query&view=query&query=GO:0005198&search_constraint=terms) | 1.89e-09 | 102 out of 808 genes, 12.6% | N18 *vs*. N2 |
| 2 | GO:0003735 | [structural constituent of ribosome](http://amigo.geneontology.org/cgi-bin/amigo/go.cgi?action=query&view=query&query=GO:0003735&search_constraint=terms) | 1.56e-07 | 58 out of 808 genes, 7.2% |  |
| 3 | GO:0003723 | [RNA binding](http://amigo.geneontology.org/cgi-bin/amigo/go.cgi?action=query&view=query&query=GO:0003723&search_constraint=terms) | 8.39e-05 | 95 out of 808 genes, 11.8% |  |
| 4 | GO:0016491 | [oxidoreductase activity](http://amigo.geneontology.org/cgi-bin/amigo/go.cgi?action=query&view=query&query=GO:0016491&search_constraint=terms) | 0.00088 | 113 out of 808 genes, 14.0% |  |
| 5 | GO:0030023 | [extracellular matrix constituent conferring elasticity](http://amigo.geneontology.org/cgi-bin/amigo/go.cgi?action=query&view=query&query=GO:0030023&search_constraint=terms) | 0.00248 | 5 out of 808 genes, 0.6% |  |
| 6 | GO:0097493 | [structural molecule activity conferring elasticity](http://amigo.geneontology.org/cgi-bin/amigo/go.cgi?action=query&view=query&query=GO:0097493&search_constraint=terms) | 0.00248 | 5 out of 808 genes, 0.6% |  |
| 7 | GO:0000182 | [rDNA binding](http://amigo.geneontology.org/cgi-bin/amigo/go.cgi?action=query&view=query&query=GO:0000182&search_constraint=terms) | 0.00028 | 4 out of 220 genes, 1.8% | S2 *vs.* N2 |
| 8 | GO:0003696 | [satellite DNA binding](http://amigo.geneontology.org/cgi-bin/amigo/go.cgi?action=query&view=query&query=GO:0003696&search_constraint=terms) | 0.00064 | 4 out of 220 genes, 1.8% |  |
| 9 | GO:0005198 | [structural molecule activity](http://amigo.geneontology.org/cgi-bin/amigo/go.cgi?action=query&view=query&query=GO:0005198&search_constraint=terms) | 6.97e-09 | 54 out of 323 genes, 16.7% | N18 *vs.* N10 |
| 10 | GO:0042302 | [structural constituent of cuticle](http://amigo.geneontology.org/cgi-bin/amigo/go.cgi?action=query&view=query&query=GO:0042302&search_constraint=terms) | 6.92e-07 | 16 out of 323 genes, 5.0% |  |
| 11 | GO:0030023 | [extracellular matrix constituent conferring elasticity](http://amigo.geneontology.org/cgi-bin/amigo/go.cgi?action=query&view=query&query=GO:0030023&search_constraint=terms) | 1.40e-05 | 5 out of 323 genes, 1.5% |  |
| 12 | GO:0097493 | [structural molecule activity conferring elasticity](http://amigo.geneontology.org/cgi-bin/amigo/go.cgi?action=query&view=query&query=GO:0097493&search_constraint=terms) | 1.40e-05 | 5 out of 323 genes, 1.5% |  |
| 13 | GO:0005214 | [structural constituent of chitin-based cuticle](http://amigo.geneontology.org/cgi-bin/amigo/go.cgi?action=query&view=query&query=GO:0005214&search_constraint=terms) | 1.83e-05 | 13 out of 323 genes, 4.0% |  |
| 14 | GO:0005201 | [extracellular matrix structural constituent](http://amigo.geneontology.org/cgi-bin/amigo/go.cgi?action=query&view=query&query=GO:0005201&search_constraint=terms) | 0.00020 | 11 out of 323 genes, 3.4% |  |
| 15 | GO:0008010 | [structural constituent of chitin-based larval cuticle](http://amigo.geneontology.org/cgi-bin/amigo/go.cgi?action=query&view=query&query=GO:0008010&search_constraint=terms) | 0.00393 | 9 out of 323 genes, 2.8% |  |
| 16 | GO:0048407 | [platelet-derived growth factor binding](http://amigo.geneontology.org/cgi-bin/amigo/go.cgi?action=query&view=query&query=GO:0048407&search_constraint=terms) | 0.00836 | 5 out of 323 genes, 1.5% |  |
| 17 | GO:0030023 | [extracellular matrix constituent conferring elasticity](http://amigo.geneontology.org/cgi-bin/amigo/go.cgi?action=query&view=query&query=GO:0030023&search_constraint=terms) | 8.64e-06 | 5 out of 290 genes, 1.7% | S 18 *vs.* N18 |
| 18 | GO:0097493 | [structural molecule activity conferring elasticity](http://amigo.geneontology.org/cgi-bin/amigo/go.cgi?action=query&view=query&query=GO:0097493&search_constraint=terms) | 8.64e-06 | 5 out of 290 genes, 1.7% |  |
| 19 | GO:0005201 | [extracellular matrix structural constituent](http://amigo.geneontology.org/cgi-bin/amigo/go.cgi?action=query&view=query&query=GO:0005201&search_constraint=terms) | 0.00063 | 10 out of 290 genes, 3.4% |  |
| 20 | GO:0048407 | [platelet-derived growth factor binding](http://amigo.geneontology.org/cgi-bin/amigo/go.cgi?action=query&view=query&query=GO:0048407&search_constraint=terms) | 0.00524 | 5 out of 290 genes, 1.7% |  |
| 21 | GO:0051082 | [unfolded protein binding](http://amigo.geneontology.org/cgi-bin/amigo/go.cgi?action=query&view=query&query=GO:0051082&search_constraint=terms) | 0.00624 | 11 out of 290 genes, 3.8% |  |
