# Supplementary material for: Global Transcriptome Sequencing Reveals Molecular Profiles of Summer Diapause Induction Stage of Onion Maggot, Delia antiqua (Diptera: Anthomyiidae)
Source: G3 (Bethesda). 2017 Nov 20;8(1):207–17. doi: 10.1534/g3.117.300393 (PMC5765349; doi:10.1534/g3.117.300393)
Supplement: Supplementary file 4 [file 207TableS4.docx]

**Table S4 Genes involved in Environmental adaption and Immune system by annotation of *D. antiqua* transcriptome and DEG analysis (FDR<=0.001, |log2Ratio|>=1)**

| **Gene ID** | **Putative Physiological Process** | **Relative gene expression level (log_2_ ratio)** | | | | | | | | | |
| --- | --- | --- | --- | --- | --- | --- | --- | --- | --- | --- | --- |
|  |  | **Symbol** | **S18/N18** | **S2/N2** | **S10/N10** | **N10/N2** | **N18/N10** | **N18/N2** | **S10/S2** | **S18/S10** | **S18/S2** |
|  | ***Circadian rhythms*** |  |  |  |  |  |  |  |  |  |  |
| CL4974.Contig3_All | casein kinase I isoform epsilon-like | *CK1ε* | -- | -- | -- | -- | 15.1 | 15.3 | -- | -- | -- |
| Unigene4102_All | casein kinase II | *CK2* | -- | 3.1 | -- | -- | -- | 2.4 | -- | -- | -- |
| Unigene10193_All | period | *per* | 8.6 | -- | 3.4 | -- | -- | -6.9 | -- | -- | -- |
| CL1746.Contig1_All | takeout | *to* |  | -8.3 |  | -6.6 |  | -3.7 | -- | -- | -- |
| CL4974.Contig3_All | casein kinase I isoform delta-A-like isoform X3 | CSNK1D | -15.0 | -- | -- | -- | 15.1 | 15.3 | -- | -- | -- |
| CL4915.Contig1_All | shaggy | sgg | -- | -- | -- | -- | -- | 2.9 | -- | -- | -- |
| Unigene12768_All | Skp1 Cdc53 F-box complex | SCF | -15.3 | -- | -- | -- | 15.4 | 15.6 | -- | -- | -- |
|  | ***Toll and IMD pathway*** |  |  |  |  |  |  |  |  |  |  |
| Unigene19424_All | toll-like receptor 13-like | *Tlr13* | -4.0 | -- | -- | -- | -- | -- | -- | -- | -- |
| CL3195.Contig2_All | Cactus | Cact | -- | -2.6 | -- | -- | -- | -- | -- | -- | -- |
| >CL3074.Contig2_All | Defensin | Def | 7.4 | -- | -- | -- | -- | -- | -- | -- | -- |
| Unigene5809_All | Ankyrin | *Ank* | -- | -3.3 | -- | -- | -- | -- | -- | -- | -- |
| CL5785.Contig6_All | JRA | *Jra* | -- | -2.6 | -- | -3.5 | -- | -- | -- | -- | -- |
|  | [***Longevity regulating pathway***](http://www.genome.jp/kegg-bin/show_pathway?map=hsa04211&show_description=show) |  |  |  |  |  |  |  |  |  |  |
| CL3790.Contig1_All | histone acetyltransferase p300-like | EP300 | -- | 2.7 | -- | -- | -- | 2.5 | -- | -- | -- |
| CL1234.Contig1_All | 4E-binding protein 2 | Eif4ebp2 | 3.4 | -- | -- | -- | -4.2 | -- | -- | -- | -- |
| CL2985.Contig2_All | 5'-AMP-activated protein kinase | AMPK | -14.6 | -- | -- | -- | 14.7 | 14.9 | -- | -- | -- |
| Unigene4265_All | Forkhead box protein O | FOXO | -- | 2.7 | -- | -- | -- | -- | -- | -- | -- |
| Unigene7788_All | Cu/Zn superoxide dismutase | SOD1 | -15.9 | -- | -- | -- | 16.0 | 16.2 | -- | -- | -- |
