# Supplementary material for: Global Transcriptome Sequencing Reveals Molecular Profiles of Summer Diapause Induction Stage of Onion Maggot, Delia antiqua (Diptera: Anthomyiidae)
Source: G3 (Bethesda). 2017 Nov 20;8(1):207–17. doi: 10.1534/g3.117.300393 (PMC5765349; doi:10.1534/g3.117.300393)
Supplement: Supplementary file 5 [file 207TableS5.docx]

**Table** **S5 Relative expression levels of various genes of selected signaling pathways in sensitive stages of non- and summer-diapause**

| **Process** | **Gene ID** | **Homologues** | **Symbols** | **S18/N18** | **S2/N2** | **S10/N10** | **N10/N2** | **N18/N10** | **N18/N2** | **S10/S2** | **S18/S10** | **S18/S2** |
| --- | --- | --- | --- | --- | --- | --- | --- | --- | --- | --- | --- | --- |
| **MAPK signaling**  **pathway** | CL1511.Contig2_ALL | Growth factor receptor-binding protein 2 | *GRB2* | -- | -15.1 | -- | -- | -- | -- | -- | -- | -- |
|  | Unigene11156_All | Guanine nucleotide-binding protein subunit alpha-12 | *G12* | -15.3 | -- | -- | -- | 15.4 | 15.6 | -- | -- | -- |
|  | Unigene10001_All | Ras-related protein Rab-40 | *Rab40C* | -- | -2.6 | -- | -- | -- | -- | -- | -- | -- |
|  | CL2358.Contig1_All | Ras-related protein Rab-5C | *Rab5C* | -14.9 | -- | **--** | **--** | **15.0** | **15.3** | **--** | **--** | **--** |
|  | CL3638.Contig1_All | Inositol-3-phosphate synthase | *ISYNA* | -15.2 | -- | **--** | **--** | **15.4** | **15.6** | **--** | **--** | **--** |
|  | CL19.Contig3_All | Cyclin-dependent kinases regulatory subunit | *CKS* | **--** | **-3.9** | **--** | **-3.9** | **--** | **-2.4** | **--** | **--** | **--** |
|  | CL1792.Contig3_All | U6 snRNA-associated Sm-like protein LSm6 | *LSM6* | -4.1 | **--** | **--** | **--** | **3.5** | **2.4** | **--** | **--** | **--** |
|  | CL928.Contig3_All | Denticleless | *DTL* | 3.9 | **--** | **--** | **--** | **--** | **--** | **--** | **--** | **--** |
| **NOTCH signaling**  **pathway** | Unigene13077_All | NOTCH |  | **--** | **-3.1** | -- | **--** | **--** | **--** | **--** | **--** | **--** |
|  | CL5727.Contig2_All | Mastermind | *mam* | -- | **2.4** | **--** | **--** | **--** | **2.6** | **--** | **--** | **--** |
|  | CL5955.Contig9_All | Deltex | *dx* | **--** | **5.2** | -- | -- | -- | 3.4 | -- | -- | -3.7 |
|  | Unigene39778_All | serrate RNA effector molecule homolog isoform X4 | *SRRT* | **--** | **5.0** | -- | -- | -- | -- | -- | -- | -5.0 |
| **VEGF signaling**  **pathway** | Unigene18156_All | serine/threonine-protein phosphatase 6 regulatory ankyrin repeat subunit B | *PP6-ARS-B* | **--** | **-3.3** | -- | **--** | **--** | **--** | **--** | **--** | **--** |
|  | Unigene8425_All | Calnexin | *Cnx* | **--** | **-3.2** | -- | **--** | **--** | **--** | **--** | **--** | **--** |
| **Calcium signaling pathway** | CL4918.Contig1_All | Adenine nucleotide translocator | *ANT* | **-15.9** | **--** | -- | -- | 16.0 | 16.3 | -- | -- | -- |
|  | Unigene19780_All | Calmodulin-like protein 4 | *CALM* | **--** | **-2.4** | **--** | **--** | **--** | **--** | **--** | **--** | **--** |
|  | CL2985.Contig2_All | Calcium/calmodulin-dependent protein kinase | *CAMK* | **-14.6** | **--** | -- | -- | 14.7 | 14.9 | -- | -- | -- |
|  | CL3790.Contig2_All | E1A/CREB-binding protein | E1A/CREBP protein | **--** | **2.3** | -- | -- | -- | -- | -- | -- | -- |

**Induction (FDR<=0.001, |log2Ratio|>=1)**
