# Supplementary material for: Global Transcriptome Sequencing Reveals Molecular Profiles of Summer Diapause Induction Stage of Onion Maggot, Delia antiqua (Diptera: Anthomyiidae)
Source: G3 (Bethesda). 2017 Nov 20;8(1):207–17. doi: 10.1534/g3.117.300393 (PMC5765349; doi:10.1534/g3.117.300393)
Supplement: Supplementary file 6 [file 207TableS6.docx]

**Table** **S6 Enzymes involved in lipid metabolism by annotation of *D. antiqua* transcriptome and DEG analysis (FDR<=0.001, |log2Ratio|>=1)**

| **Gene ID** | | **Putative Physiological Process** | **Relative gene expression level (log_2_ ratio)** | | | | | | | | | | |
| --- | --- | --- | --- | --- | --- | --- | --- | --- | --- | --- | --- | --- | --- |
|  |  |  | **Symbol** | **S18/N18** | **S2/N2** | **S10/N10** | **N10/N2** | **N18/N10** | **N18/N2** | **S10/S2** | **S18/S10** | **S18/S2** | |
|  | | ***Steroid biosynthesis*** |  |  |  |  |  |  |  |  |  |  |  |
| CL967.Contig1_All | | Delta14-sterol reductase | ERG24 | -- | -3.46 | -- | -- | -- | -- | -- | -- | -- |  |
| CL721.Contig1_All | | sterol-4alpha-carboxylate 3-dehydrogenase | NSDHL | **-**14.8 | -- | -- | -- | 15.0 | 15.2 | -- | -- | -- |  |
| Unigene17684_All | | Fatty acid synthase | fas-1 | **--** | -3.22 | -- | -- | -- | -- | -- | -- | -- |  |
| CL1935.Contig1_All | | triacylglycerol lipase |  | **-0.77** | -4.99 | -2.90 | -0.405 | -2.47 | -2.82 | 1.67 | -0.30 | 1.38 |  |
|  | | ***Ether lipid metabolism*** |  |  |  |  |  |  |  |  |  |  |  |
| Unigene877_All | phospholipase D1/2 | PLD1_2 | -16.6 | **--** | **--** | -- | 16.7 | 16.9 | -- | -- | -- |  |  |
|  | | ***Glycerophospholipid metabolism*** |  |  |  |  |  |  |  |  |  |  |  |
| CL4983.Contig4_All | | aldehyde dehydrogenase (NAD+) | ALDH | 4.7 | -- | -- | -- | -3.3 | -- | -- | -- | -- |  |
|  | | ***Arachidonic acid metabolism*** |  |  |  |  |  |  |  |  |  |  |  |
| CL4602.Contig1_All | | secretory phospholipase A2 | PLA2G | -- | -5.68 | -- | -- | -- | -15.9 | -- | -- | -- |  |
|  | | ***alpha−Linolenic acid metabolism*** |  |  |  |  |  |  |  |  |  |  |  |
| Unigene8249_All | | acyl-CoA oxidase | ACOX1 | 4.9 | -- | -- | -- | -- | -- | -- | -- | -- |  |
|  | | ***Cutin, suberine and wax biosynthesis*** |  |  |  |  |  |  |  |  |  |  |  |
| Unigene71_All | | alcohol-forming fatty acyl-CoA reductase | FAR | -- | -2.54 | -- | -- | -- | -2.6 | -- | -- | -- |  |
|  | | ***Linoleic acid metabolism*** |  |  |  |  |  |  |  |  |  |  |  |
| CL3700.Contig4_All | | butanol dehydrogenase | bdhAB | 4.0 | -- | -- | -- | -- | -- | -- | -- | -- |  |
|  | | ***Sphingolipid metabolism*** |  |  |  |  |  |  |  |  |  |  |  |
| CL2307.Contig4_All | | alkaline ceramidase | ASAH3 | -- | -3.67 | -- | -- | -- | -- | -- | -- | -- |  |
| Unigene16461_All | | acid ceramidase | ASAH1 | -15.1 | -- | -- | -- | 15.2 | 15.5 | -- | -- | -- |  |
|  | | ***Fatty acid metabolism*** |  |  |  |  |  |  |  |  |  |  |  |
| Unigene1446_All | | acetyl-CoA C-acetyltransferase | atoB | -15.6 | -- | -- | -- | 15.7 | 15.9 | -- | -- | -- |  |
| Unigene9190_All | | alcohol dehydrogenase | ADH1_7 | -15.5 | -- | -- | -- | 15.6 | 15.8 |  |  |  |  |
| Unigene17141_All | | aldehyde dehydrogenase | ALDH | -15.7 | -- | -- | -- | 15.8 | 16.1 | -- | -- | -- |  |
