# Supplementary material for: Global Transcriptome Sequencing Reveals Molecular Profiles of Summer Diapause Induction Stage of Onion Maggot, Delia antiqua (Diptera: Anthomyiidae)
Source: G3 (Bethesda). 2017 Nov 20;8(1):207–17. doi: 10.1534/g3.117.300393 (PMC5765349; doi:10.1534/g3.117.300393)
Supplement: Supplementary file 7 [file 207TableS7.docx]

**Table S7 Enzymes involved in Carbohydrate metabolism by annotation of *D. antiqua* transcriptome and DEG analysis (FDR<=0.001, |log2Ratio|>=1)**

| **Gene ID** | **Putative Physiological Process** | **Relative gene expression level (log_2_ ratio)** | | | | | | | | | |
| --- | --- | --- | --- | --- | --- | --- | --- | --- | --- | --- | --- |
|  |  | **Symbol** | **S18/N18** | **S2/N2** | **S10/N10** | **N10/N2** | **N18/N10** | **N18/N2** | **S10/S2** | **S18/S10** | **S18/S2** |
|  | ***Citrate cycle (TCA)*** |  |  |  |  |  |  |  |  |  |  |
| Unigene500_All | citrate synthase | CS | -- | -3.26 | -- | -- | -- | -- | -- | -- | -- |
| CL5120.Contig1_ALL | isocitrate dehydrogenase | IDH3 | -- | -2.93 | -- | -- | -- | -- | 3.9 | -- | -- |
| CL2155.Contig1_All | succinyl-CoA synthetase | LSC1 | -- | 4.90 | -- | -- | -- | 4.8 | -- | -- | -- |
| CL2990.Contig1_All | ATP citrate (pro-S)-lyase | ACLY | -15.9 | -- | -- | -- | 16.0 | 16.2 | -- | -- | -- |
| Unigene18720_All | aconitate hydratase | ACO | -15.3 | -- | -- | -- | 15.4 | 15.7 | -- | -- | -- |
| Unigene7628_All | isocitrate dehydrogenase | IDH | -15.6 | -- | -- | -- | 15.7 | 16.0 | -- | -- | -- |
|  | ***Amino sugar and nucleotide sugar metabolism*** |  |  |  |  |  |  |  |  |  |  |
| Unigene15212_All | mannose-6-phosphate isomerase | manA | -- | -5.0 | -- | -- | -- | -- | -- | -- | -- |
| CL2492.Contig1_All | mannose-1-phosphate guanylyltransferase | GMPP | **--** | 14.4 | -- | -- | -- | -- | -- | -- | -- |
| CL5011.Contig2_All | chitinase | Chia | 8.1 | -- | 7.7 | -- | -- | -- | -- | -- | -- |
| Unigene1703_All | UDPglucose 6-dehydrogenase | UGDH | -- | -- | 7.6 | -- | -- | -- | -- | -- | -- |
