# Supplementary material for: Global Transcriptome Sequencing Reveals Molecular Profiles of Summer Diapause Induction Stage of Onion Maggot, Delia antiqua (Diptera: Anthomyiidae)
Source: G3 (Bethesda). 2017 Nov 20;8(1):207–17. doi: 10.1534/g3.117.300393 (PMC5765349; doi:10.1534/g3.117.300393)
Supplement: Supplementary file 8 [file 207TableS8.docx]

**Table S8 Enzymes involved in Energy metabolism by annotation of *D. antiqua* transcriptome and DEG analysis (FDR<=0.001, |log2Ratio|>=1)**

| **Gene ID** | **Putative Physiological Process** | **Relative gene expression level (log_2_ ratio)** | | | | | | | | | |
| --- | --- | --- | --- | --- | --- | --- | --- | --- | --- | --- | --- |
|  |  | **Symbol** | **S18/N18** | **S2/N2** | **S10/N10** | **N10/N2** | **N18/N10** | **N18/N2** | **S10/S2** | **S18/S10** | **S18/S2** |
|  | ***Oxidative phosphorylation*** |  |  |  |  |  |  |  |  |  |  |
| CL5516.Contig3_All | F-type H+-transporting ATPase | ATPeF0C | -- | 3.5 | -- | -- | -- | -- | -- | -- | -- |
| CL4152.Contig1_All | V-type H+-transporting ATPase | ATPeV1E | -- | -2.5 | -- | -- | -- | -- | -- | -- | -- |
| Unigene21307_All | F-type H+-transporting ATPase | ATPeF1D | **--** | -- | -15.1 | 15.2 | -15.4 | -- | -- | -- | -- |
| Unigene15563_All | cytochrome c oxidase | COX6B | **--** | -- | -4.2 | 3.1 | -4.1 | -- | -- | -- | -- |
| CL1487.Contig2_All | ubiquinol-cytochrome c reductase cytochrome | fbcH | 14.7 | -- | -- | -- | -14.8 | -14.1 | -- | -- | -- |
| Unigene8863_All | cytochrome c oxidase cbb3-type | ccoN | 16.9 | -- | -- | -- | -16.9 | -16.7 | -- | -- | -- |
| Unigene10148_All | NADH dehydrogenase | DHase | 14.8 | -- | -- | -- | -14.5 | -14.0 | -- | -- | -- |
| CL5478.Contig2_All | Inorganic pyrophosphatase | PPA | -15.6 | -- | -- | -- | 15.7 | 15.9 | -- | -- | -- |
| Unigene19622_All | NADH-ubiquinone oxidoreductase chain 5 | ND5 | 14.7 | -- | -- | -- | -15.0 | -14.6 | -- | -- | -- |
| Unigene19964_All | F-type H+-transporting ATPase | ATPeF0A | 15.2 | -- | -- | -- | -15.2 | -14.3 | -- | -- | -- |
| CL547.Contig1_All | V-type H+-transporting ATPase | ATPeV0C | -14.8 | -- | -- | -- | 14.9 | 15.1 | -- | -- | -- |
| CL3964.Contig2_All | cytochrome c oxidase | COX3 | 15.7 | -- | -- | -- | -15.5 | -15.5 | -- | -- | -- |
| Unigene8863_All | cytochrome c oxidase | COX1 | 16.9 | -- | -- | -- | -17.0 | -16.7 | -- | -- | -- |
| Unigene12006_All | cytochrome c oxidase | COX2 | 14.5 | -- | -- | -- | -14.5 | -14.2 | -- | -- | -- |
| CL4030.Contig3_All | cytochrome c oxidase assembly protein | COX17 | 4.1 | -- | -- | -- | -- | -- | -- | -- | -- |
| Unigene7788_All | superoxide dismutase Cu-Zn | SOD | -15.9 | -- | -- | -- | 16.0 | 16.2 | -- | -- | -- |
| CL624.Contig3_All | 14-3-3-like protein | 14-3-3 | -14.7 | -- | -- | -- | 14.8 | 15.0 | -- | -- | -- |
| CL1539.Contig2_All | electron-transferring-flavoprotein dehydrogenase | ETFD | -- | -3.2 | -3.9 | -- | -- | -1.9 | -- | -- | -- |
| CL250.Contig2_All | ATP synthase | ATP5D | -- | -- | -4.5 | 4.3 | -- | -- | -- | -- | -- |
| Unigene510_All | Cytochrome c oxidase assembly protein COX11 | COX11 | -- | -3.5 | -- | -- | -- | -- | -- | -- | -- |
|  | ***stress response*** |  |  |  |  |  |  |  |  |  |  |
| CL615.Contig2_All | Heat shock protein 67B3 | Hsp67B3 | -- | -7.2 | -- | -- | -- | -- | 7.1 | -- | -- |
| CL492.Contig1_All | 23kDa heat shock protein ScHSP23 | Hsp23 | -- | -5.0 | -- | -- | -- | -- | 5.3 | -- | -- |
| Unigene13292_All | Heat shock protein 67B2 | Hsp67B2 | -- | -4.1 | -- | -- | -- | -- | -- | -- | -- |
| CL2164.Contig2_All | Heat shock protein 90 | Hsp90 | -16.1 | -- | -- | -- | 16.2 | 16.5 | -- | -- | -- |
| CL2973.Contig1_All | heat shock protein 70 | Hsp70 | -16.2 | -- | -- | -- | 16.3 | 16.5 | -- | -- | -- |
| CL3803.Contig1_All | heat shock protein 20 | Hsp20 | -15.5 | -- | -- | -- | 15.6 | 15.8 | -- | -- | -- |
| CL5130.Contig1_All | 60 kDa heat shock protein | Hsp60 | -14.9 | -- | -- | -- | 15.0 | 15.2 | -- | -- | -- |
| CL538.Contig1_All | Heat shock protein 100 | Hsp100 | -15.2 | -- | -- | -- | 15.3 | 15.6 | -- | -- | -- |
| Unigene1058_All | heat shock protein 16-like | Hsp16 | -15.4 | -- | -- | -- | 15.5 | 15.8 | -- | -- | -- |
| Unigene11209_All | Small heat shock protein hspI | HspI | -15.6 | -- | -- | -- | 15.7 | 16.0 | -- | -- | -- |
| Unigene11255_All | 17.9 kDa class I heat shock protein | Hsp17.9 | -16.3 | -- | -- | -- | 16.4 | 16.6 | -- | -- | -- |
| Unigene12150_All | 97 kDa heat shock protein | Hsp97 | -16.1 | -- | -- | -- | 16.2 | 16.4 | -- | -- | -- |
| Unigene12150_All | heat shock protein 110kDa | Hsp110 | -16.1 | -- | -- | -- | 16.2 | 16.4 | -- | -- | -- |
| Unigene17621_All | heat shock protein 16-like | Hsp16 | -15.6 | -- | -- | -- | 15.7 | 15.9 | -- | -- | -- |
| Unigene18707_All | Heat shock protein 83 | Hsp83 | -14.6 | -- | -- | -- | 14.8 | 15.0 | -- | -- | -- |
| Unigene9606_All | 10 kDa heat shock protein | Hsp10 | -14.7 | -- | -- | -- | 14.8 | 15.0 | -- | -- | -- |
| CL5922.Contig3_All | glutathione S-transferase | GST | 3.5 | -- | -- | -- | -- | -- | -- | -- | -- |
| CL842.Contig1_All | Ferritin | Ferritin | -- | 2.3 | -- | -- | -- | -- | -- | -- | -- |
| CL1751.Contig2_All | Cytochrome P450 | CYP450 | -- | -6.0 | -- | -- | -- | -- | -- | -- | -- |
| Unigene14320_All | [peroxiredoxin](https://en.wikipedia.org/wiki/Peroxiredoxin) | Prdx | -15.2 | -- | -- | -- | 15.3 | 15.5 | -- | -- | -- |
| Unigene10864_All | Catalase | Cata | -15.6 | -- | -- | -- | 15.7 | 15.9 | -- | -- | -- |
